# Adapting in polycrisis: Ensuring access to land as a polysolution for Arctic reindeer herding

**DOI:** 10.1007/s13280-026-02379-x

**Published:** 2026-04-19

**Authors:** Simo Sarkki, Sirpa Rasmus, Antti-Juhani Pekkarinen, Mikko Jokinen, Ilona Mettiäinen, Tuuli Parviainen, Laura Post, Taru Rikkonen, Jaana Sorvali, Tuulia Väärälä, J. Otto Habeck, Jussi T. Eronen

**Affiliations:** 1 Cultural Anthropology, University of Oulu, P.O. Box 1000, 90014 Oulu, Finland; 2 Natural Resources Institute Finland (Luke), Paavo Havaksen Tie 3, 90570 Oulu, Finland; 3 Arctic Centre, University of Lapland, P.O. Box 122 (Pohjoisranta 4), 96101 Rovaniemi, Finland; 4 Ecosystems and Environment Research Programme, Faculty of Biological and Environmental Sciences, University of Helsinki, P.O. Box 65 (Viikinkaari 1), 00014 Helsinki, Finland; 5 Natural Resources Institute Finland (Luke), Latokartanonkaari 9, 00790 Helsinki, Finland; 6 Natural Resources Institute Finland (Luke), Ounasjoentie 6, 96200 Rovaniemi, Finland; 7 Helsinki Institute of Sustainability Science (HELSUS), Faculty of Biological and Environmental Sciences, University of Helsinki, P.O. Box 65 (Viikinkaari 1), 00014 Helsinki, Finland; 8 University of Applied Sciences of Lapland, Jokiväylä 11C, 96300 Rovaniemi, Finland; 9 Institute for Social and Cultural Anthropology, Universität Hamburg, Edmund-Siemers-Allee 1, 20146 Hamburg, Germany; 10 BIOS Research Unit, Meritullintori 6 A 14, 00170 Helsinki, Finland

**Keywords:** Climate change, Green transition, Indigenous peoples and local communities, Land use, Nature-based livelihoods, Shared Socioeconomic Pathways

## Abstract

Current global socio-environmental changes challenge human societies and give rise to new adaptation needs also for Indigenous Peoples and Local Communities (IPLCs). Based on participatory workshops with Sámi and Finn reindeer herders we examined adaptation challenges for reindeer herding in a situation characterized by complex, intertwined and unpredictable risks framed as polycrisis. We found that increasing the possibilities of reindeer herders to influence the land-use governance to ensure their access to land can help them to cope with several challenges linked to green transition, geopolitics, inequality, and climate change simultaneously. Access to land thus functions as a practical “polysolution”. Furthermore, based on inductive analysis of the workshop discussions, we identified five criteria to understand polysolutions: depth, width, pace, proactiveness, and collaboration. These criteria can also help to identify solutions for other IPLCs and their nature-based livelihoods facing similar unpredictable and complex challenges that necessitate adaptation in polycrisis.

**Supplementary Information:**

The online version contains supplementary material available at 10.1007/s13280-026-02379-x.

## Introduction

Current global socio-environmental changes challenge human societies and pose novel needs for adaptation. Recent major environmental assessments have emphasized the need to recognize the knowledge, values, rights, cultures, and histories of Indigenous Peoples and Local Communities (IPLCs), whose well-being is compromised Sarkki & Rasmus as joint first authors. By crises related to climate change (IPCC 2022), biodiversity loss, and unbalanced use of ecosystem services (IPBES 2019; UN 2022). In addition, the COVID-19 pandemic (Fitzmaurice 2021), economic crises (UN 2010), and conflicts over land and resources (Mowforth 2014) are influencing realities of various IPLCs. A reindeer herder noted in a recent participatory workshop that *“nowadays it feels that after one has lived through one crisis, the next challenge awaits”* (Rasmus et al. 2023). These notions motivated us to analyse the case of Arctic reindeer herding, assuming that the livelihood is facing outcomes of the global polycrisis.

Reindeer herding depends on natural conditions (weather, pasture environment), collaboration (herding work, pasture use-related negotiations), and markets. This means it is vulnerable to various kinds of intertwined, interacting, and rapidly evolving developments. Various hazards and risks can lead to crisis—in the crisis situation, the viability of a livelihood or other system is threatened. Crisis means “approaching a tipping point”, after which the system either is transformed (change in the identity and characteristics of the livelihood) or even collapses if there is no way to adapt anymore (e.g. Landauer et al. 2021). The situation has been described using terms cumulative impacts (e.g. Stoessel et al. 2022), cumulative worries (Bostedt et al. 2025), multiple stressors (Hovelsrud et al. 2020), compound impacts (Harnesk et al. 2023), and cascading impacts (Paulsen et al. 2023). Polycrisis—a key concept in our work—is closely related to these previously formulated concepts. The situation reindeer herders are facing can be considered as an outcome of global polycrisis, especially because of increasing complexity and emerging surprises. The global polycrisis occurs *“when crises in multiple global systems become causally entangled in ways that significantly degrade humanity’s prospects”* (Lawrence et al. 2022). A polycrisis can be experienced on a local scale, for example in Arctic nature-based livelihoods facing complex and intertwined impacts of global developments. Mitigation of polycrisis is difficult if not impossible, exposure to future hazards unavoidable, and thus, strong emphasis is placed on adaptation (Tooze 2022; Hoyer et al. 2023). Leading from this, we propose that *adaptation in polycrisis* means adjustments of existing policies, economies, socio-cultural systems, and human–environment interactions in ways that reduce vulnerability towards unexpected future crises. Ways to respond to the grand adaptation challenge of polycrisis are yet unknown, but crucial in understanding current and future developments of human societies.

We examine how the outcome of the global polycrisis for the IPLCs occurs at the intersection of climate crisis, land-use change related to the “green transition”, and geopolitical tensions, through the case of reindeer herding in Finland. The livelihood is practised by both Indigenous Sámi and ethnic Finn local communities (Sarkki et al. 2021; Rasmus et al. 2023). Reindeer herding depends on herders’ access to land and availability of natural pastures, and its prerequisites are largely determined by seasonal climatic conditions. It is thus strongly affected by global environmental change (Horstkotte et al. 2022). It has been estimated that climate change is 2–4 times faster in the Arctic than the global average (Rantanen et al. 2022). In addition, 85% of the pasture areas for reindeer and sheep in Fennoscandia are affected by at least one other land-use pressure and 60% by multiple pressures (Stoessel et al. 2022). Nature conservation can also cause pressures: many IPLCs could be severely impacted if ambitious conservation targets would be implemented denying access of IPLCs to their home areas and resources needed for their livelihoods (Schleicher et al. 2019). Furthermore, the green transition can be unjust for Arctic IPLCs, as it increases land-use forms like wind power development and mining projects (Normann 2021; Nilsson and Sarkki 2022). The starting point for this paper is that reindeer herding is simultaneously being impacted by multiple cumulative hazards and risks challenging the continuity of the livelihood (Horstkotte et al. 2022). Thus, the situation of reindeer herding necessitates adaptation measures that can function as polysolutions, i.e. solutions which can effectively address multiple challenges simultaneously (Mark et al. 2025). However, case studies and more detailed criteria for polysolutions are urgently needed to respond to the grand adaptation challenge in the era of the global polycrisis.

Our objective is to examine the outcomes of the global polycrisis as manifested in reindeer herding, and based on the inductive analysis, to propose criteria for understanding polysolutions. Our research questions are: (1) what are the priority elements in the operational environment of reindeer herding, (2) what are the challenges in reindeer herding, imposed by intertwined and simultaneously occurring developments linked to various Shared Socioeconomic Pathways (SSPs), (3) what kind of adaptation measures are used and proposed, to cope with these challenges, and 4) what kind of criteria can be identified for polysolutions.

## The key concepts and the case of reindeer herding

### Polycrisis and shared socioeconomic pathways

Polycrisis is defined as “multiple co-occurring, causally entangled crises with synergistic and cascading effects on multiple systems” (Rakowski et al. 2025), a situation threatening the viability of the systems in question (Lawrence et al. 2023; Henig and Knight 2023). Polycrisis challenges the traditional adaptive measures that target each crisis individually (Mark et al. 2025), as polycrisis refers to a systemic crisis, distinguishing it from sudden and isolated disruptions (Lawrence 2024). Polycrisis emphasizes the complexity of the systems in which the risk develops instead of the complexity of the risks (Lawrence et al. 2023). It also acknowledges that solutions to certain challenges can lead to new risks. According to Tooze (2022), humanity has been adapting to and coping with the past crises rather successfully—for instance, we have avoided nuclear war, invented vaccines, and dodged great depressions—but solutions rarely address the underlying trends, leading to increased tensions. Furthermore, polycrisis arises from interactions among multiple systems, increasing the probability of actualization of risks across systems. Hence, “*polycrisis is not offering a new, different type of disaster; rather, it is raising the spectre that these disasters … will tend to extend beyond any single system and spatial or political boundary*” (Mark et al. 2025). There is a strong systemic focus in the concept of polycrisis. Still, power and agency also play a key role in responding to polycrisis (Lawrence 2024). The global polycrisis can manifest itself in various ways in different localities (Rakowski et al. 2025). To make sense of how the global polycrisis is manifested in reindeer herding in Finland, we use four key questions to untangle the complex concept (Lawrence 2024) (Table 1).

**Table 1:** Key questions to understand the outcomes of the global polycrisis in the case of reindeer herding in Finland

Key questions to understand polycrisis (Lawrence 2024)	Examples on the links to the reindeer herding case
What is the relevant system, where are its boundaries, and how does it relate to other systems?	Finnish reindeer herding area is divided into 54 Reindeer Herding Cooperatives (RHC). Their operational environment shares many issues linked to herders’ rights, climate change, increase in other land uses, and knowledge and values of herders. However, situations differ for example between the 13 Sámi RHCs in the northernmost Finland and the other RHCs lying south of Sápmi
What range of states and behaviours constitutes the normal functioning of this system?	Reindeer herding has traditionally relied on natural pastures for subsistence of reindeer (Landauer et al. 2021; Åhman et al. 2022), and there is a strong intergenerational element as most new herders are children of herders
What feedbacks once maintained an established equilibrium, and why did they cease to do so?	Herders’ access to land has maintained the livelihood system, but this is challenged by increase in other land uses, climate change, and predator conservation (Pape and Löfler 2012; Landauer et al. 2021). The recent increase in geopolitical tensions and emphasis on self-sufficiency in minerals and energy impact herding, e.g. through pressures for mining projects (Joona 2025) and wind power development in a way that may undermine minority rights of herders (Nystén-Haarala et al. 2021; Eftestøl et al. 2023)
What events and behaviours indicate that a system is reaching atipping point (i.e. in a systemic crisis)?	Catastrophic winter and summer conditions have been experienced due to the accelerating climate change (Rasmus et al. 2022); geopolitical tensions shift policy priorities (Vinkel 2023), predator-caused reindeer losses are high (RHA 2025), supplementary feeding of reindeer is increasing to cope with difficult winter conditions, loss of winter pastures to forestry and predation pressure (Åhman et al. 2022); intergenerational continuity of reindeer herding is at risk (Markkula et al. 2024; Sarkki et al. 2025)

We approach this polycrisis reindeer herding is facing by utilizing the Shared Socioeconomic Pathways (SSPs) narratives related to future climates and socioeconomic developments (e.g. O’Neill et al. 2020). The SSP scenario set includes five narratives: SSP1: Sustainability (Taking the Green Road), SSP2: Business-As-Usual (Middle of the Road), SSP3: Regional Rivalry (A Rocky Road), SSP4: Inequality (A Road Divided), and SSP5: Fossil-Fuelled Development (Taking the Highway) (O’Neill et al. 2017; Riahi et al. 2017). In terms of the relevance of these future narratives for reindeer herding, green transition links to SSP1, geopolitical tensions brought forth especially by the Russian aggression against Ukraine since 2022 link to SSP3, perceived inequalities link to SSP4, and accelerating climate change in the Arctic link to SSP5. We use these topics as starting points to understand the current situation in reindeer herding. The SSPs are well known and widely applied, and thus, connecting our case to them can increase the generalizability of our findings to the cases of other IPLCs. The intertwined SSPs are a way to approach the global polycrisis; here we focus on outcome of the global polycrisis faced by reindeer herders in Finland.

### Critical impacts and reindeer herding as a livelihood

The development trajectories listed above have varying impacts on local realities. Reindeer herding is a traditional nature-based livelihood (Table 1). While herding is an economic activity, it is also a way of life, and, especially for Indigenous Sámi herders, a culturally important profession. In addition, herding is a transgenerational occupation that is commonly passed on from one generation to the next. We approach the reindeer herding livelihood system as an operational environment consisting of environmental, governance, economics, and socio-cultural elements and interconnections (Horstkotte et al. 2022; Sarkki et al. 2022a; Rasmus et al. 2023). Furthermore, we assume that some of these are more critical than others, in terms of their intrinsic values for livelihood practitioners and also in terms of their instrumental function in helping to cope with a polycrisis.

### Adaptation in reindeer herding

Adaptation in reindeer herding is most often discussed in relation to climate change. As the IPCC defines it, adaptation in this context means planning and actions to “prepare for and adjust to the current and projected impacts of climate change”. The idea is to minimize the harmful impacts of changing conditions and utilize the potential benefits (IPCC 2012). Adaptation is very often reactive, coping (Noble et al. 2014; Rasmus et al. 2020), as is the case with supplementary feeding (Table 1). This means responding to a short-lived event, an effort that herders employ in their work to minimize the damage to their livelihood due to unfavourable conditions. Proactive adaptation, on the other hand, means adjustments based on deliberate decisions and long-term planning, even when certain climate change impacts (or other pressures) are not yet observed. Proactive adaptation calls for preparedness and anticipation but also requires understanding the elements of desired futures and emphasizing actions towards those (Noble et al. 2014).

Relevant concepts in adaptation are *hazard*, *exposure*, *vulnerability* and *risk* (IPCC 2012). Risks build up when vulnerable systems are exposed to hazards—events or developments possibly causing harm or danger. Reindeer herding is increasingly exposed to various hazards and harms caused by other land uses, climate change, growing predator populations, and socioeconomic developments (already in Pape and Löffler 2012). Vulnerability of the livelihood can be reduced through well-planned adaptation. However, herding is forced to adapt in ways that are not often beneficial for the livelihood (Reinert et al. 2009; Huntington et al. 2019; Fohringer et al. 2021; Rosqvist et al. 2022a; O'Faircheallaigh 2023). As a response, proposals for a holistic approach to adaptation have been made (Hovelsrud et al. 2020)—planning and actions that enable responding to a multitude of changes in a sustainable way. A high adaptive capacity is needed. Adaptive capacity is defined as a potential to moderate potential damage, to utilize the opportunities, or to manage the consequences (Smit and Wandel 2006; Noble et al. 2014). Elements of adaptive capacity in reindeer herding are many, for example anticipation and preparedness, diversifying the livelihood, communication, flexibility in pasture use, traditional knowledge, education and skills, and well-functioning support from the governance level (Ford et al. 2016; Rasmus et al. 2022).

Adaptation takes place and is governed at various levels. There is practical adaptation—planning and actions—at herder, family and community level (Rasmus et al. 2020). Policies and rules at regional, national, and even international governance levels (institutional adaptation) support, or in some cases limit, the everyday level of practical adaptation. Adaptation is often siloed, which means that actors plan and act separately. Coordination of the needs of various sectors and land uses is missing—an element that would be needed in holistic, transformative adaptation (Lonsdale et al. 2015).

There are limits to adaptation: reindeer physiology and behaviour, as well as geography of the herding district and other land uses within its boundaries, impose limits, reducing flexibility in pasture use (Risvoll and Hovelsrud 2016; Tyler et al. 2021; Löf et al. 2022). Certain adaptation actions may be impossible because of lack of resources, and lack of power to influence institutional decision-making processes is also often mentioned (Löf 2013; Rasmus et al. 2022; Rosqvist et al. 2022a). According to Löf et al. (2022), the present-day governing systems across Fennoscandia related to reindeer herding are not sufficient to support the livelihood when it faces cumulative and interacting hazards. The situation diverges depending on whether the livelihood is challenged by individual stressors, calamity events, cumulative impacts, or a polycrisis (Table 2).

**Table 2:** Overview of types of risks faced in reindeer herding, with examples of possible adaptation

Type of risks	Reindeer herding example	Scale	Event occurrences	Adaptation examples
Individual stressors	Individual competing land uses leading to pasture encroachment	Spotted, very local	Continuous	Reconciliation of land uses (e.g. reindeer herding—forestry conflict in Inari; Raitio 2008; Sarkki et al. 2022b)
Calamity events	Extreme weather conditions; significant predator damages	The whole reindeer herding area but with regional variation	Randomly sequential	Compensations for losses (Heikkinen et al. 2011); supplementary feeding, climate catastrophe fund (Rasmus et al. 2022)
Cumulative impacts	Several land-use developments in one RHC	Specific Reindeer Herding Cooperatives	Continuous, simultaneous	Holistic community-led impact assessments (Rosqvist et al. 2022b)
Polycrisis	Simultaneous occurrence of all the above	The whole reindeer herding area	Continuous, sequential, simultaneous	Polysolutions

Polycrisis calls for new types of adaptive solutions. Hence, the concept of polysolution has been proposed referring to (1) strategies that have benefits across different systems and that address multiple issues simultaneously rather than treating different crises in isolation (Mark et al. 2025); (2) integrated approaches that address the root causes of interlinked crises (Schreiber et al. 2025); and (3) solutions addressing the target issue, while simultaneously generating positive outcomes in neighbouring economic, social and ecological domains (Bohn et al. 2025).

## Materials and methods

### Empirical material

**Fig. 1:**
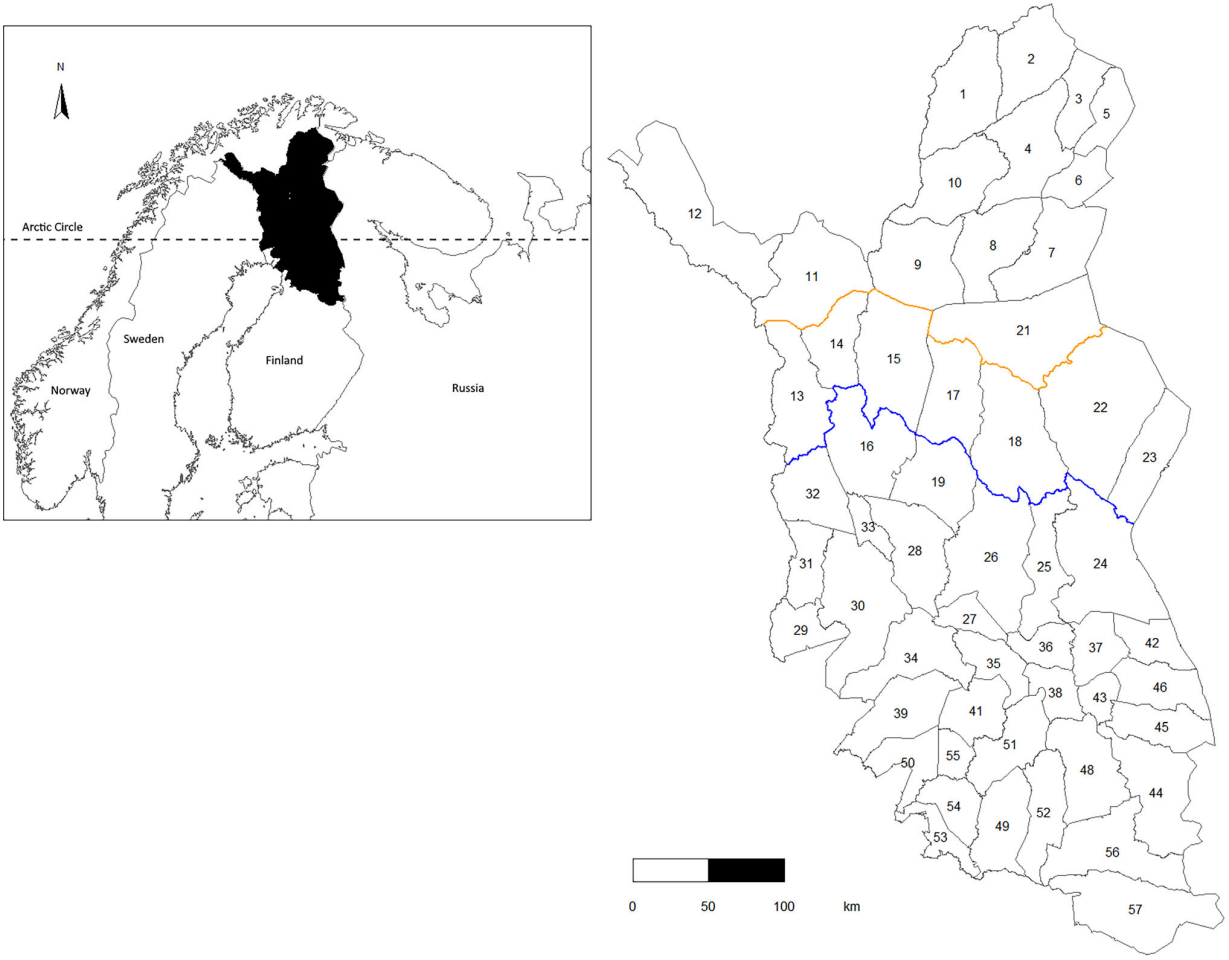
Reindeer herding area in Finland. Yellow line is the southern border of the Sámi homeland area, and the blue line is the southern border of the area specially intended for reindeer herding. Numbers refer to reindeer herding cooperatives (https://paliskunnat.fi/reindeer-herders-association/cooperatives/). Blue circles mark the workshop locations, Inari (northern one) and Rovaniemi (southern one). Map: Arto Vitikka; adopted from Rasmus et al. 2021

In Finland, reindeer herding constitutes a traditional nature-based livelihood with a long history both for Sámi and also for ethnic Finn herders (e.g. Kortesalmi 2008; Horstkotte et al. 2022). The reindeer herding area (RHA) is divided into 54 reindeer herding cooperatives (RHCs, Fig. 1). Thirteen northernmost RHCs belong to the Sámi homeland area in Finland.

To examine the current issues in reindeer herding we arranged two participatory futures workshops in 2022, first one in Inari, and second one in Rovaniemi, Finland (Fig. 1). The first workshop was organized in connection to “Reindeer and Fish Research Days”^1^, and the invitation to the workshop was sent to all participants of these days. In addition to organizers, there were 27 participants in the workshop; they represented seven RHCs, out of which four were located in Sámi homeland area. In addition to herders, participants were from Reindeer Herders’ Association, Sámi Parliament, Sámi RHCs’ association, Suoma Boazosamit Association, local and regional authorities (municipality, Centre for Economic Development, Transport and the Environment of Lapland), research organizations, and agriculture and forestry interest organization of Finland. The first workshop took place in four breakout groups (for the workshop report see Rasmus et al. 2023; in Finnish, summary in English). In the second workshop, there were 12 participants from Reindeer Herders’ Association’s board, consisting mostly of leaders of RHCs around different regions within the RHA. The second workshop was conducted in two breakout groups (both workshops discussed in Pekkarinen and Rasmus 2024).

Both workshops utilized a similar method with slightly diverging applications (details in Wang et al. 2025; Rasmus et al. 2025). The workshop included three sessions. The first session was about identifying the most important elements of the operational environment of reindeer herding. Each participant could select three cards from the pre-prepared deck of 52 cards; it was also possible to add elements not found on the deck of cards. Each card described an element linked either to (a) pastures and land use, (b) reindeer herding practices and herders, (c) climate, environment and reindeer as animals, and d) economy, society, and governance. The elements on the cards had originally been selected by the authors. After this, the deck of cards had been refined during a co-creative iteration process involving for example herders and students of nature-based livelihoods (Wang et al. 2025; Rasmus et al. 2025). This prioritization led to a set of approximately 20 cards in each breakout group. Then the participants constructed cognitive maps of the operational environment of reindeer herding based on the chosen elements, in each breakout group. Cognitive maps are one way to bring a systems perspective into workshop discussions by identifying key elements of the system and their relationships (e.g. Goodier and Soetanto 2013). Cognitive maps can aid in the understanding of complex systems and even identify implications of specific policy and governance options (Kok 2009).

The second session considered what will happen to the constructed operational environment under the influence of a major driver. First, “what if” questions, connected to the global SSP narratives (O’Neill et al. 2017; Riahi et al. 2017), were formulated by researchers. We did not seek to downscale the global narratives but rather used them to inform identification of “what if” questions relevant for reindeer herding that are also linked to global developments. Four “what if” questions considered were discussed in the first workshop: (1) acceleration of climate change and its implications for reindeer herding (linking to SSP5); (2) intensifying green transition and its land-use related impacts especially through increasing wind power, mining and predator conservation (linking to SSP1); (3) the continued Russian war of aggression against Ukraine since 2022 and its relevance for reindeer herding (linking to SSP3); and (4) influence of pandemics, such as COVID-19, on reindeer herding. The groups discussed and modified their cognitive maps based on the discussions on “what if” questions (the second workshop was shorter, and we included these major drivers as cards in the original deck where participants chose the priority elements). Even when structural inequalities (linking to SSP4) were not introduced as a major driver in either of the workshops, the topic was widely discussed.

In the third session, participants identified their livelihood-related aims and dreams, and the most critical points of the operational environment for the realization of these. The participants first wrote down their individual dreams and then placed arrows on the cognitive maps (the second workshop was concluded by a joint discussion on short-term and long-term proposals on how the governance of the livelihood could enhance the realization of these aims and dreams).

The workshop method was designed based on a combination of insights from literature on futures workshop methods and the authors’ long-term participatory work with reindeer herders. The facilitation was flexible and the questions were open ended: the aim was to learn about concerns and solutions from the participants, also outside the topics prepared beforehand. According to feedback, participants considered the workshop method productive, while hoping that future workshops would also include decision-makers and other land users as participants, to nurture cross-sectoral dialogue (Rasmus et al. 2025).

### Analysis methods

Extensive notes were taken by organizers from both of the workshops. All cognitive maps were photographed, and the prioritization of the elements and the aims and dreams were documented. The written materials were analysed by qualitative directed content analysis (Hsieh and Shannon 2005). The materials from the first workshop were used to create an extensive report (Rasmus et al. 2023). The second workshop was conducted to strengthen the results. In general, we found that results from both workshops were in line with each other, yet with diverging details (Pekkarinen and Rasmus 2024).

To identify and understand the possible polysolutions for reindeer herding, we used a four-step analysis. First, we considered that a polysolution strengthens the critical elements in the operational environment of reindeer herding (Sect. "Priority elements and the operational environment of reindeer herding"). To identify these priority elements, we used the insights gained from the first session in both workshops, and discussions on livelihood-related dreams and aims. Second, to verify that reindeer herding is facing outcomes of the global polycrisis we analysed the insights mainly from the first workshop considering the SSPs and their relevance for reindeer herding (see Sect. "Shared socioeconomic pathways and reindeer herding"). Third, we identified existing and proposed adaptation measures for responding to individual challenges. We clustered the adaptation measures discussed under the SSPs (see Sect. "Adapting to individual crises separately"). Fourth, to identify general criteria to assess what kind of adaptation measures can be considered as polysolutions we used inductive content analysis. Five criteria were identified by going through the workshop results and seeking answers to the question of what kind of measures can enhance the continuity of reindeer herding in the midst of a polycrisis. We clustered the 79 individual findings, leading to the formulation of five general criteria that characterize polysolutions (see Sect. "Criteria for polysolutions").

## Results

### Priority elements and the operational environment of reindeer herding

In both workshops, the most often mentioned aim of herders was that the livelihood would continue for the future generations in one’s own family. Such continuity was linked to the priority elements of the operational environment, forming the essence of the reindeer herding livelihood. Based on the prioritization exercise in both workshops, eight priority elements were identified in all breakout groups. Furthermore, in one of the breakout groups, values and identity, communality among herders, and traditional practice-based knowledge were considered to form “the soul” of the livelihood (workshop 1). In the workshop 2, winter pastures, pasture rotation, and using targeted feeding as a tool to control the herds (*paimennusruokinta*) were considered as the core of reindeer herding. Indigenous rights, customary rights to herd the reindeer, and communality among herders were considered as “treasures” of the herding culture.

During the session 3, participants marked the most critical spot in the cognitive maps for the realization of their livelihood-related dreams and aims. Land-use planning was the most frequently marked spot in workshop 1. This is supported by the quotations from the workshop 2:*“Land-use planning and recognition of herders’ rights influence the whole livelihood”.* The importance of land-use governance links to herders’ access to land, which was frequently discussed in both workshops. Furthermore, the question is not only about access, or pasture conditions, but about “grazing peace”—meaning that reindeer can move about and forage according to their needs, not being harassed by predators or distracted by human activities. Disturbance caused by other land uses and infrastructure also deteriorates the grazing peace. Participants themselves added grazing peace as a new priority element in workshop 2. Economy and well-being of herders were considered as crucial and directly linked to the motivation of the next generation to continue to work in reindeer herding. Communality was also seen as a key dimension of the livelihood, linking especially to practical herding tasks. Local knowledge was considered as an important element that has developed in a close connection between herders in many generations, reindeer, and pasturelands. Our interpretation of this discussion is that herders’ access to land connects to all priority elements (Table 3).

**Table 3:** Eight elements in the operational environment of reindeer herding prioritized in all breakout groups in both workshops linking to the continuity of the livelihood workshops, and their clustering by authors

Priority elements identified in all breakout groups in both workshops	Clusters of priority elements	Generalized themes related to the continuity of the livelihood
Land-use planning	Participation in the land-use governance	Pastures and land use
Weather and snow conditions	Available pastures	
Winter pastures		
	Grazing peace (Added element based on workshop 2)	
Reindeer herds and welfare of reindeer	Welfare of reindeer	Reindeer herding communities
Profitability and costs	Economy and well-being of reindeer herders	
Well-being of reindeer herders		
Collaboration and communality	Communality	
Traditional knowledge and know-how	Local knowledge	

### Shared socioeconomic pathways and reindeer herding

**Fig. 2:**
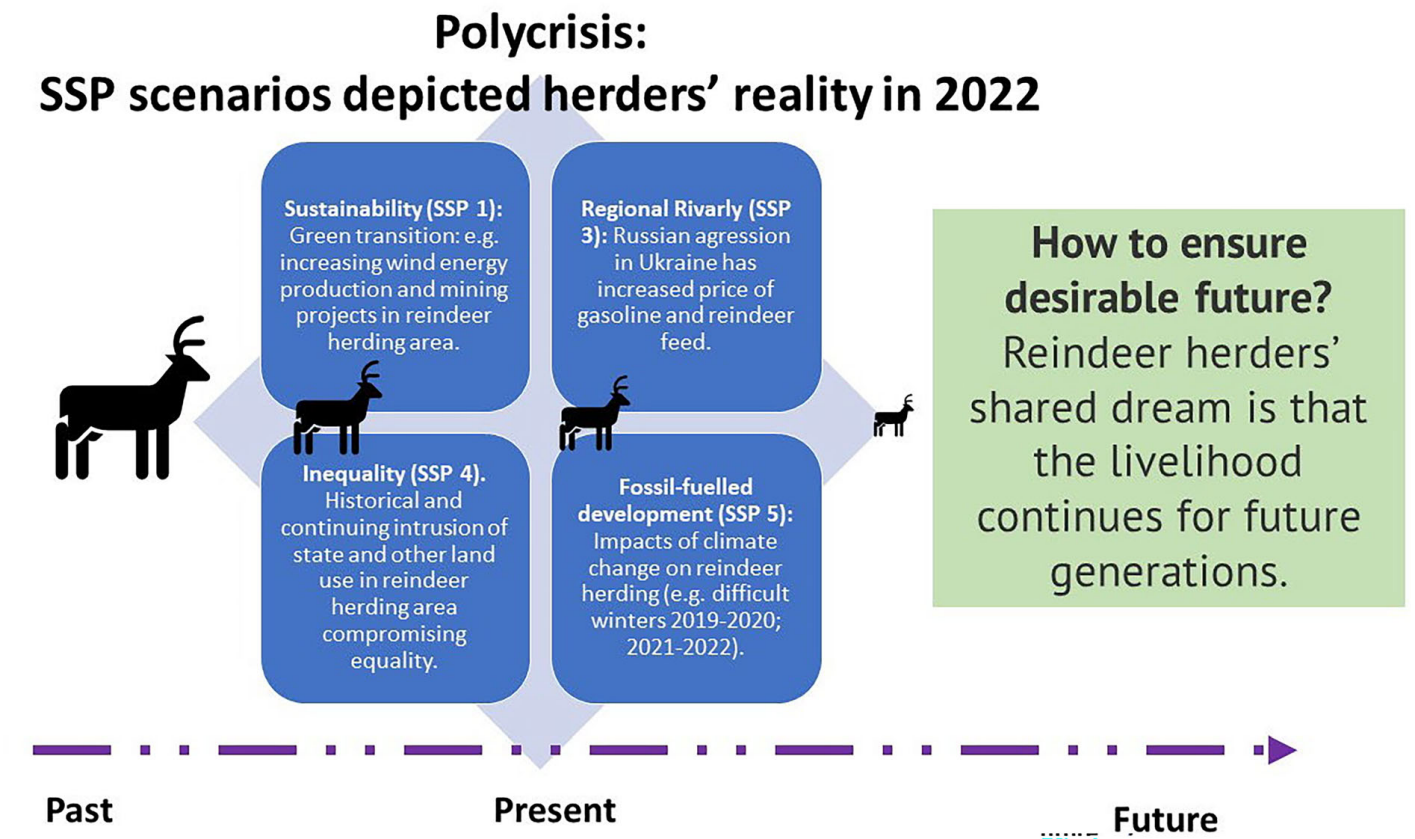
Multiple interlinked crises experienced in reindeer herding, and linking of these to SSP narratives

The results from both workshops pointed towards multiple crises challenging the continuity of the livelihood for next generations. In the workshop 1, the participants discussed accelerating climate change (linking to SSP5), increasing land-use pressures related to green transition (linking to SSP1), unstable geopolitical situation (linking to SSP3), and the COVID-19 pandemic. The overall conclusion from these discussions was that the development trajectories depicted by various SSP narratives are not mutually exclusive and do not describe distant future, but current reality. Inequality (linking to SSP4) emerged as a topic in both workshops, for example in discussions related to externally led land-use developments on reindeer pastures, and to herders’ limited power to influence these. In the workshop 2, participants considered that the continuity of reindeer herding is challenged by various pressures including climate change and the rising costs of fuel and feed due to current geopolitical tensions. In both workshops, it became clear that the crises are interlinked (Fig. 2).

SSP5 was seen to connect to the increased frequency of extreme weather events like heat waves and difficult snow conditions, during which certain pastures are required, meaning that climate change also links to access to land. Furthermore, climate change was considered to influence the welfare of reindeer through new diseases and pests, and changes in the growth of lichen—an important forage for reindeer.

SSP1 was seen as linked to the green transition, increasing land-use pressures influencing herders’ access to land, especially through mining and wind power projects. The green transition was also considered as connected to economy and well-being of herders through potentially decreasing tourism, demands to give up the use of motorized vehicles in herding tasks, and pressure to decrease reindeer numbers to help lichen pastures to recover. Increasing number of predators, affecting both reindeer welfare and well-being of herders, was also seen as connected to SSP1.

SSP3 was connected to geopolitical tensions and the impacts of the war in Ukraine on Europe, which had increased the price of fuel and supplementary reindeer feed, thus affecting herders’ economy. The increasing need to become self-sufficient in energy production and critical minerals was considered as a potential threat to access to land and to herders’ opportunities to participate in land-use related decisions. SSP4 was seen to be linked to concrete land-use developments leading to encroachment of pastures and to cumulative impacts on reindeer herding (Table S1).

### Adapting to individual crises separately

The results from both workshops implied that the dominant idea of adaptation needs to be revisited to deal with an ongoing polycrisis situation in reindeer herding and to ensure the well-being of herders and the welfare of reindeer. It is the herder who adapts, but with a diverging degree of support from governmental actors. Table S2 identifies examples of herders’ adaptive measures towards risk and crises linked to individual SSPs, based on workshop discussions. Many of the reactive measures are already in use, but some of them, like the catastrophe fund or rapid emergency feeding with the state support, and most of the listed proactive measures are still plans and recommendations or aims and dreams of herders. Implementation of these measures would need action and resources both from the state level, and at the local level. For example, in the workshop 2, participation fatigue was recognized among herders who have to participate in multiple land-use development processes within their RHC. It was felt that in order to avoid participation fatigue, governmental organizations could carry out more holistic planning, acknowledge the cumulative impacts within RHCs, and bring actors together to look at various land-use developments simultaneously. Ensuring herders’ access to land and opportunities to participate in land-use related decision-making were at the centre of the adaptive measures discussed in both workshops regarding all SSPs, and as such could function as polysolutions.

### Criteria for polysolutions

To identify general criteria for polysolutions, we identified a set of 79 individual statements voiced in the workshops that inform preferable adaptation measures. We inductively categorized the statements under 16 sub-criteria, further generalized under five general criteria for polysolutions: depth, width, pace, proactiveness, and collaboration (Table S3).

The first criterion, the depth of change, highlights that polysolutions should address the causes of the reindeer herding livelihood’s vulnerability to a polycrisis. The identified sub-criteria highlight that such vulnerability can be decreased by holistic recognition of values linked to reindeer herding, by securing grazing peace of reindeer, by enhancing herders’ opportunities to participate in the land-use planning, and by proper recognition of herders’ rights, including Indigenous Sámi rights. The workshop discussions highlighted the impacts of other land use on pastures and reindeer (session 1, all breakout groups). Land use was also linked to the continuity of reindeer herding identity and culture (session1, especially group 1), and passing this on to future generations who would still have the possibility to live with reindeer (session 3, all breakout groups). This would be enabled by good-quality natural pastures (session 3, all breakout groups).

The second criterion, the width of change, links to cumulative impacts of other land uses on reindeer herding, but also other hazards and risks, forming a local manifestation of the global polycrisis. The overall results highlighted that reindeer herding is facing challenges occurring not only from one, but from several trends depicted by various SSPs (Sect. "Shared socioeconomic pathways and reindeer herding"). Another sub-criterion was to connect adaptation measures of reindeer herding to other policies and policy objectives, such as supporting biodiversity and ecological restoration, green transition, or equality and inclusion.

The third criterion, the pace of solutions, highlights the urgency of adaptation measures. This criterion was formed based on three sub-criteria. The first one highlights the connection between the welfare of reindeer and the well-being of herders (session 1, breakout group 2); these can both be enhanced by enhancing herders’ opportunities to influence the land-use related decision-making (session 3, all breakout groups). The second one emphasizes the urgent need to improve profitability, to enhance the continuity of livelihood. The third one links to chronic crisis facing the livelihood—sometimes life of a herder “feels like going from one crisis to the next” (herder in the workshop 1). Hence, urgent solutions are needed to enhance the coping capacity. Workshop discussions stressed the importance of fast measures to emerging crises, for example caused by extreme whether events. This could mean developing compensation systems and establishing a catastrophe fund (session 2, especially breakout group 4). However, solutions were asked also for coping with the long-term encroachment of reindeer pastures due to other land use (session 1, breakout group 2). The more the solutions are postponed the more extensive other land use will be, leading to deterioration and loss of reindeer pastures.

The fourth criterion, proactiveness, stresses the need to anticipate future challenges instead of reactive attempts to cope with emerging ones. Sub-criteria link to proactive adaptation to climate change and to the use of court cases as means to influence land-use planning. Predation-caused reindeer losses were also discussed. Reactive compensations cover the economic losses for herders, but justify prioritization of predator populations over reindeer, leading to adverse situation where “reindeer do not anymore feed people, but herders feed predators” (herder in the workshop 2).

The fifth criterion, collaboration, highlights the engagement in the design of adaptation measures. Sub-criteria link to trust building, need to reconcile between different land users, resources available for collaboration, herders’ communality, and recognition of herders’ traditional knowledge. It was widely agreed in the workshops that adaptation should not be solely the responsibility of herders, since they have not caused the problems (all groups). However, land-use planners and decision-makers are not experts in reindeer herding. Participation in planning processes by herders is therefore necessarily needed, but this burdens and takes time away from herding tasks (session 1, breakout group 3). Herders’ opportunities to influence land-use related decision-making need to be enhanced (all groups) with the help of extra resources to participate (session 1, breakout group 3). Furthermore, mutual understanding, trust and well-functioning collaboration between different land users was hoped for (session 3, all groups).

## Discussion

**Fig. 3:**
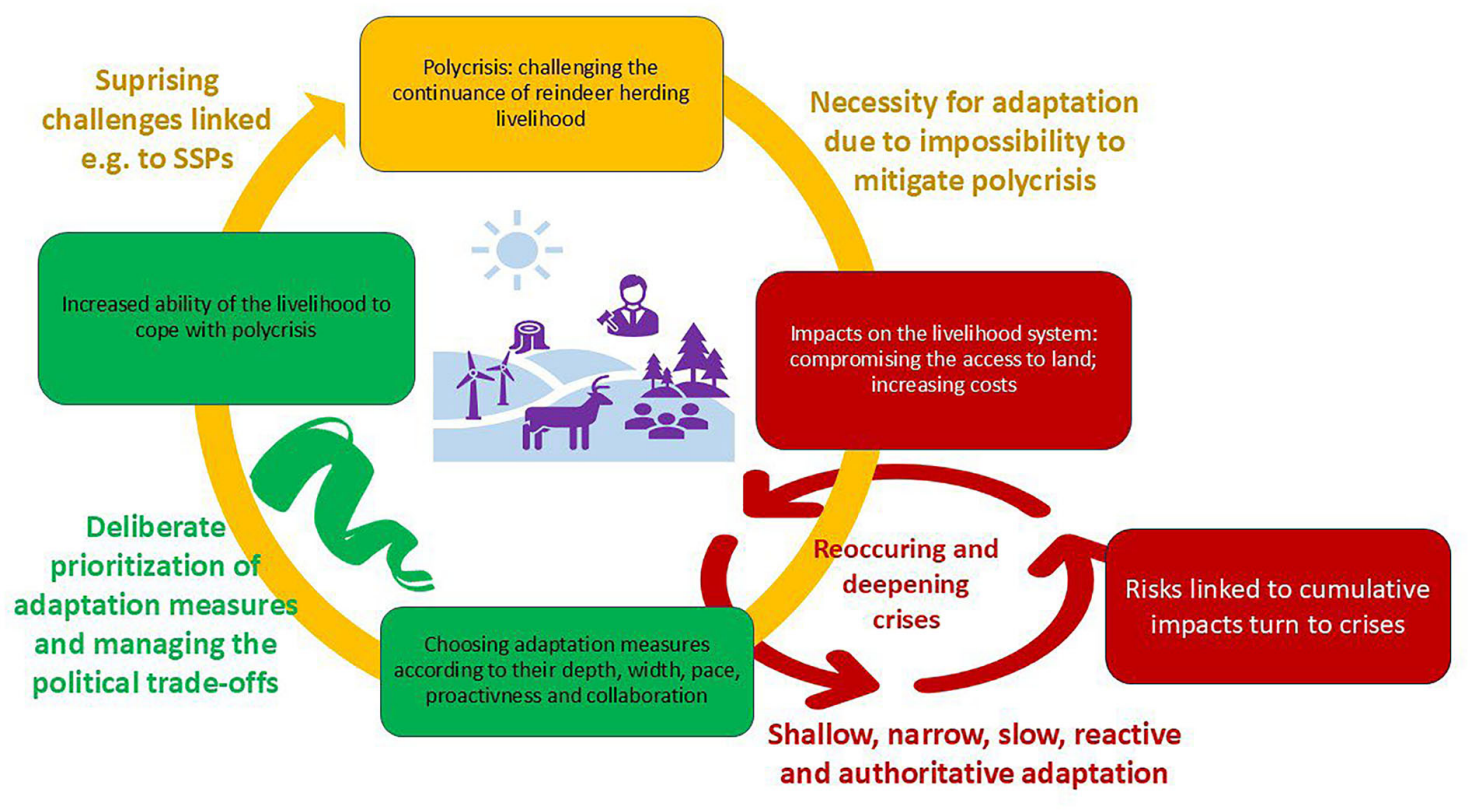
Livelihoods in polycrisis: key characteristics of our emerging framework. The framework starts from the yellow box considering that polycrisis emerges as a continuous set of surprises that together challenge the continuity is a livelihood. Due to the complexity, polycrisis cannot be mitigated, and hence adaptation is crucial. To adapt eff ectively, the five criteria of depth, width, pace, proactiveness and collaboration need to be considered. Yet, often the adaptation measures need to be prioritized and also political tensions linked to the livelihood and other relevant actors need to be transparently addressed. The employment of the criteria to prioritize adaptation measures can help to cope with polycrisis until new surprises emerge

Increasing the opportunities of herders to influence decision-making related to land use would support their meaningful adaptation in a polycrisis and can thus act as a polysolution. Based on this finding and our other results learnt from the case of reindeer herding, we discuss the five criteria for understanding polysolutions: depth, width, pace, proactiveness, and collaboration. While we identified these criteria inductively, they also have resonance with other literature on transformative change and adaptation. For example, the following underlying key dimensions for transformative adaptation have been identified: depth, scope, scale, and pace (e.g. Termeer et al. 2024; Engbersen et al. 2024). Our results highlight the relevance of these dimensions. Width in our categorization links to scope and scale, and we add criteria linked to proactive and collaborative adaptation measures; aspects earlier emphasized for example in future-oriented studies on human–environment interactions (MA 2005a, b) and in the context of climate change (Braunschweiger 2022) (Table 4). Yet, the combination of our five inductively identified criteria is unique. Figure 3 outlines our general framework deriving from the case study to understand adaptation in a polycrisis. This framework might also be relevant for understanding other local manifestations of the global polycrisis.

**Table 4:** Comparing existing criteria used in connection to transformative adaptation and transformative change to our proposed criteria for understanding polysolutions with examples from the reindeer herding case

Definition of criteria in existing literature	Proposed criteria for polysolutions (authors)	Link to polysolutions in reindeer herding case
*Depth* Level of change covering values, frames, and logics underlying the current system (Termeer et al. 2024) *Depth* Change of the system of interest involving new values, goals and beliefs (Engbersen et al. 2024)	*Depth* Adaptations that address root causes of vulnerability to a local manifestation of the global polycrisis	Adaptations that ensure reindeer herders’ access to land on which the livelihood depends on
*Scope* Large-scale, system-wide change (Termeer et al. 2024) *Scale* Change affects local, regional, national and / or supranational scale (Engbersen et al. 2024)	*Width* Adaptations that address simultaneously several symptoms of locally experienced outcomes of the global polycrisis	Adaptations that address cumulative impacts by several other land users, climate change and predator conservation together
*Pace* Rapid and accelerated response (Termeer et al. 2024) *Speed* Quick change given the scope, scale and complexity (Engbersen et al. 2024)	*Pace* Adaptations that occur fast to avoid larger damage, often increasing with time and inaction	Adaptations that can rapidly respond to emergent crises like extreme whether conditions or sudden increase in predators in certain areas
*Proactive adaptation* Anticipatory and future-oriented adaptation (Noble et al. 2014)	*Proactiveness* Adaptations that anticipate future challenges rather than reactively respond to crises	Adaptations that increase preparedness to anticipated future crises (e.g. funds for climate adaptation)
*Scope* Involving various actor groups across domains (Engbersen et al. 2024)	*Collaboration* Adaptations that are substantially reframed by those who experience a local polycrisis in collaboration with policy makers, and other key stakeholders	Adaptations linked to reframing land-use planning to ensure herders’ genuine opportunities to influence land-use decisions

### Depth of adaptation: Enhancing herders’ access to land

Depth of adaptation refers to measures that address the root causes of vulnerability (Fedele et al. 2019) to a polycrisis as it occurs in specific places and for different livelihoods. This often requires inclusion of diverse actors to reframe adaptation policies (Eriksen et al. 2015). The depth of adaptation measures can be explored by first identifying the critical elements in the operational environment of a livelihood, like reindeer herding. Our case study implies that compromised access to land by herders is the key challenge for the continuity of the livelihood—a finding also confirmed by earlier research (e.g. Risvoll and Hovelsrud 2016; Landauer et al. 2021). Access to land was frequently discussed in both workshops, and the impacts of other land use on “grazing peace” of reindeer and the availability of seasonal pastures were highlighted as key issues that impact both the welfare of reindeer and the well-being of herders, these two being tightly interlinked (Horstkotte et al. 2024). Hence, depth of adaptation can be advanced by reframing policies and land-use planning towards solutions that secure herders’ access to land, for example by securing herders’ rights in concrete land-use decisions.

### Width of adaptation: Addressing several symptoms simultaneously

Width of adaptation refers to solutions that are system-wide (Termeer et al. 2024). In the reindeer herding case, this connects to solutions that cover the whole reindeer herding area in Finland, and that can address challenges experienced differently across RHCs. The key problem here is the siloed policymaking, which leads to addressing individual challenges separately. While we identified the set of adaptive measures targeting individual SSPs and related challenges (Table S2), we consider that such adaptive measures should be prioritized that can address multiple pressures and symptoms simultaneously. Opportunities of herders to influence the land-use planning help them to adapt to climate change by enabling pasture rotation, and more flexible opportunities for pasture use during difficult weather and snow conditions (Rasmus et al. 2022) This way it offers alternatives for supplementary feeding of reindeer and thereby helps to reduce the additional costs emerging from increasing prices of feed and fuel due to geopolitical tensions. Having influence on land-use governance can help herders to cope with inequalities related to land-use developments (Junka-Aikio 2023) and rectify some experiences related to the “green colonialism” (Normann 2021; Joona 2025).

### Pace of adaptation: Addressing symptoms fast

The pace of adaptation measures is of crucial importance (Termeer et al. 2024), as future costs of policy inaction often outweigh the costs of adaptation measures if implemented today, as shown by climate change research (Sanderson and O’Neill 2020). We highlight two issues in connection to the reindeer herding case. First, the sudden and emerging symptoms of a polycrisis need to be addressed fast to avoid larger damage, often increasing with time and inaction. This is evident, for example, regarding reindeer losses due to extreme whether events (e.g. icing of pastures) and predator damages. In both cases, waiting before taking the adaptation measures—such as extensive emergency feeding (Rasmus et al. 2022) or legal hunting of predators causing damage (Heikkinen et al. 2011)—leads to significant reindeer losses. Second, the pace of adaptation links to the potential to address the underlying causes of the emergent crisis fast (‘t Hart and Boin 2001). While mitigating climate change and geopolitical negotiations are of pivotal importance, they offer resolutions slowly. Instead, tackling inequality, and pressures related to the green transition by increasing herders’ opportunities to influence the land-use planning could offer faster solutions, if there is political will to secure their traditional nature-based livelihood in Finland. The recent report of the Sámi Truth and Reconciliation Commission in Finland (2025) addressing past, present, and future injustices regarding Sámi people is a step towards this direction. The report included recommendations related to adaptation to climate change and to ensuring the continuity of traditional livelihoods like reindeer herding; however, these consider Sámi people practising herding, not the whole herding livelihood regardless of the ethnic background of practitioners.

### Proactive adaptation

Proactive adaptation is anticipatory and future-oriented, compared to reactive adaptation, which emerges only after crises occur (Noble et al. 2014). Herders highlighted in the workshops that there is a significant need to move from reactive to proactive adaptation, for example, to be better prepared for extreme weather conditions (also Rasmus et al. 2022). Furthermore, having limited power over land-use planning, herders have initiated court cases and conflicts to influence the land-use decisions (Sarkki et al. 2022b). This can be seen as a proactive move to pressure the current land-use planning system to change.

### Collaborative design of adaptation measures

Collaboration between diverse actors is seen as a cornerstone of effective and innovative adaptation to climate change (Raciti et al. 2025). Our results emphasize that herders cannot adapt effectively alone, and that the state-based adaptation planning may neglect the local perspective. In order for the state-based governance to support sustainable adaptation of reindeer herding, the governed system needs to be reimagined with a strong engagement by herders themselves (Löf et al. 2022). This means, for example, acknowledging the locally defined elements of desired futures; local aims; and dreams (Rasmus et al. 2025). Our workshop results emphasized the need for collaborative approaches in adaptation, ensuring that the know-how and local knowledge of herders is utilized. For example, it was mentioned that not all adaptation measures are possible to implement, nor culturally acceptable. Also, it was highlighted that challenges emerge when the planning sector does not understand the problems posed by other land uses on reindeer pastures. Thus, herders’ collaboration with land-use planners, and adaptation planning, is crucial. Yet, as our results implied, there is a need for additional resources for herders to participate, because participation typically burdens the same persons and takes away time from herding tasks (see Sarkki et al. 2025). Herders considered getting support for collaboration and participation as fair; herders are not themselves causing the problems and a polycrisis they are experiencing.

### Wider relevance of the findings

We conducted a case study of reindeer herding in Finland to examine how the outcomes of the global polycrisis are manifested for the traditional nature-based livelihoods of IPLCs. Our findings on intertwined development trends depicted by the SSP narratives point to increasingly complex and unpredictable challenges for reindeer herders. This suggests that other IPLCs may also be encountering diverse manifestations of the global polycrisis. Climate change is a global challenge for IPLCs (Leal Filho et al. 2021; Garai et al. 2022), intersecting with other pressures and exacerbating historical effects of social and political marginalization of IPLCs (Reyes-García et al. 2024). The green transition, or green colonialism, is not unique to the Finnish Arctic (Schmid 2023). The current geopolitical tensions have global implications (Vinkel 2023), for example, through the price of fuel and the supply of grain. The well-being and equal opportunities of IPLCs are also often constrained by structural inequalities (e.g. Humphreys Bebbington 2013).

An example from Norway illustrates the increasing recognition of Indigenous land rights in concrete policy decisions. The Norwegian Supreme Court ruled in 2021 that two wind power farms in Fosen violated the cultural rights of Sámi herders, leading to compensations and veto rights for herders for future wind farm licences (Mósesdóttir 2024). Such a court ruling is part of a continuum of court cases by the Sámi against the Norwegian state since the Alta conflict in the 1970s. While the ruling is a significant precedent, it is only a step towards full recognition of Indigenous Sámi rights to their homelands. Thus, while the Fosen decision complies with the criteria of depth and proactiveness, its width is dependent on subsequent court cases. The pace of the process has been slow, and relationships between actors are characterized rather by conflict than collaboration. This brief account shows that the usability of our five criteria is resonates with other case studies addressing similar issues.

## Conclusion

The present paper has examined how the outcomes of the global polycrisis are experienced in reindeer herding in Finland. Based on the case study, we identified five criteria for polysolutions: depth, width, pace, proactiveness, and collaboration. Previous work on polysolutions has focused on solutions that address multiple challenges simultaneously (Mark et al. 2025), address the root causes of interlinked crises (Schreiber et al. 2025), or generate positive outcomes for several neighbouring systems (Bohn et al. 2025). Our five criteria bring additional insight into the topic and, through the reindeer herding case, can also inform adaptation measures in the global polycrisis.

Our case shows that developments described by several SSPs are taking place simultaneously and are leading to frequent harms, concretized risks, and continuous surprises in reindeer herding. Polycrisis is a close relative to concepts introduced in previous studies on cumulative impacts and compounding stressors posing challenges for reindeer herding, but polycrisis as a concept is more complex, unpredictable, and globally connected. While the global polycrisis is impossible to mitigate, local adaptations are possible. Successful adaptation in a polycrisis requires polysolutions. Our major finding is that increasing the possibilities of reindeer herders to influence land-use planning is the most effective way to enhance the prospects for the continuity of the livelihood for future generations also in the face of unpredictable future challenges. The importance of herders’ genuine opportunities to participate in land-use planning has been widely established in literature during the past two decades (e.g. Hukkinen et al. 2006; Raitio 2008; Sarkki et al. 2022b). Land rights of Indigenous Peoples are linked to self-determination and cultural continuity also elsewhere (Ojong 2020; Larson et al. 2022). Yet, the present study is the first one that considers herders’ genuine opportunities for participation in land-use planning as a polysolution. It does not only address the challenges posed by competing land uses, but also (1) decreases the dependency on supplementary feeding of reindeer requiring extra resources; (2) enhances possibilities to adapt to extreme whether events by maintaining alternative pastures and enabling pasture rotation; (3) addresses persisting inequalities in a concrete way; and (4) advances the justice of green transition taking place, for example, as wind power developments and mining projects within the reindeer herding area. While there are apparent trade-offs with other land users, and a need for political will to enhance herders’ genuine participation opportunities, there are also synergies. International and national policy objectives linked to equality and social justice can be supported. Opposition to the green transition can be mitigated by making it more socially just. Reindeer herding contributes to food security and to keeping remote rural areas populated, which are also security issues. Saving diverse pastures for reindeer can support biodiversity. Hence, herders’ opportunities to influence land-use planning can act as a polysolution not only for herders, but also to deliver other societal and policy objectives. This partly explains, and also more widely legitimizes, IPLCs’ strong emphasis on the significance of their access to land.

## Supplementary Information

Below is the link to the electronic supplementary material.

Supplementary file1 (PDF 216 KB)

## Footnotes

1
https://www.luke.fi/en/projects/porokala2022.
