# Supplementary material for: Adapting in polycrisis: Ensuring access to land as a polysolution for Arctic reindeer herding
Source: Ambio. 2026 Apr 19;55(8):1768–84. doi: 10.1007/s13280-026-02379-x (PMC13319551; doi:10.1007/s13280-026-02379-x)
Supplement: Supplementary file 1 — Supplementary file1 (PDF 216 KB) [file 13280_2026_2379_MOESM1_ESM.pdf]

Supplementary Information

This Supplementary Information has not been peer reviewed.

Adapting in polycrisis: Ensuring access to land as a polysolution for Arctic reindeer herding

Table S1. Summary of the SSP narratives (Riahi et al. 2017), related actualised and anticipated risks and hazards based on workshop discussions, and priority elements impacted by the identified risks and hazards. The COVID-19 pandemic considered as a separate driver of change.

Brief description of SSP narratives	Anticipated and actualized risks and hazards
**SSP5: Fossil-Fuelled Development: Taking the Highway**<br><br>Increasing faith in competitive markets, innovation and participatory societies to produce rapid technological progress and development of human capital as the path to a sustainable development.	**Acceleration of climate change**<br>- Increasingly frequent extreme whether events including storms and heat waves (Welfare of reindeer)<br>- Unpresented snow conditions like very deep snow cover (Available pastures)<br>- Increasingly frequent icing of pastures, and icy layers in the snow (Available pastures)<br>- Dry and hot summers (Welfare of reindeer)<br>- Reindeer diseases, pests, and insects (Welfare of reindeer)<br>- Changes in the growth of ground and tree lichen and mushroom (Welfare of reindeer)
**SSP1: Sustainability: Taking the Green Road**<br><br>The world shifts toward a more sustainable path, emphasizing more inclusive development that respects perceived environmental boundaries**.**	**Land-use pressures related to green transition**<br>- Increase of land-use developments (especially renewable energy production like wind power) (Available pastures; Grazing peace; Participation)<br>- Increase of mining projects to meet increasing global mineral demand (Grazing peace;; Participation)<br>- Pressure to reduce the use of gasoline-based motor vehicles in daily herding practices (Economy and well-being of herders)<br>- Decrease of international tourism and thus decreasing opportunities for herders to get income from tourism related products and services (Economy and well-being of herders)<br>- Decrease of number of reindeer herders (Economy and well-being of herders)<br>- High numbers of predators and losses of reindeer due to predation (Welfare of reindeer; Economy and well-being of herders).

**SSP3: Regional Rivalry: A Rocky Road**<br><br>A resurgent nationalism, concerns about competitiveness and security, and regional conflicts push countries to increasingly focus on domestic or, at most, regional issues.	**Continuance of the war situation in Europe**<br>- Increase in price of reindeer feed (Economy and well-being of herders).<br>- Increase in price of fuel (Economy and well-being of herders).<br>- Increased need for energy production in domestic setting that can interfere with reindeer herding (Available pastures; Grazing peace; Participation).).
**SSP4: Inequality: A Road Divided**<br><br>Highly unequal investments in human capital, combined with increasing disparities in economic opportunity and political power, lead to increasing social stratification both across and within countries	**Increasing land use in reindeer herding area**<br>- Increase in cumulative impacts of land use; further colonialisation of the North by external actors intensifies (Participation; Available pastures; Grazing peace).).<br>- Participation fatigue of reindeer herders due to increasing amount of land-use planning processes where it would be essential to try to influence (Participation).
**The COVID-19 pandemic**	- Loss of customers for reindeer meat, need to search for new channels to sell the meat (Economy and well-being of herders).<br>- Decrease of price of reindeer meat by large customers (Economy and well-being of herders).<br>- Decrease in the number of tourists in reindeer herding area (Economy and well-being of herders).<br>- Difficulties in arranging the herding tasks within family if everyone is sick (Communality; Welfare of reindeer; Economy and well-being of herders).<br>- Changes in the collaborative herding work and social interactions (Communality)<br>- New future risks for infectious diseases between animals and people (Welfare of reindeer and well-being of herders).

References:

Riahi, K., Detlef P. van Vuuren, Elmar Kriegler, Jae Edmonds, Brian C. O'Neill, Shinichiro Fujimori, Nico Bauer, Katherine Calvin, Rob Dellink, Oliver Fricko, Wolfgang Lutz, et al. 2017. The Shared Socioeconomic Pathways and their energy, land use, and greenhouse gas emissions implications: An overview. Global Environmental Change 42: 153-168. https://doi.org/10.1016/j.gloenvcha.2016.05.009.

Table S2. Adaptive measures towards risk and crises linked to individual SSPs, based on workshop discussions. Measures are clustered under reactive and proactive adaptation (clustering by authors).

**SSP1**	**Adaptive measures based on workshop discussions**
Reactive adaptation measures	- Subsidies and compensations to increase social equality in nature conservation and green transition (e.g. increasing wind power and mining) (Participation; Economy and well-being of herders)).<br>- Taking advantage of increased potential synergies between reindeer herding and protected areas (only if reindeer are allowed within protected areas) (Grazing peace, Available pastures) .<br>- Compensations for losses due to predation, losses needed to be proven after damages have taken place (Economy and well-being of herders; Welfare of reindeer).
Proactive adaptation measures	- Considering the role of reindeer herders in restoring ecosystems alongside the EU nature restoration law (Participation).<br>- Promoting the idea of biocultural diversity that should be safeguarded instead of narrow biodiversity (Participation; Available pastures)).<br>- Ensuring Indigenous Sámi rights, and rights to herd reindeer (Participation; Available pastures) .<br>- Green certification for reindeer meat (Economy and well-being of herders).<br>- Rapid and straightforward processes to grant licenses to hunt predators causing severe damage (Economy and well-being of herders; Welfare of reindeer).
**SSP3**	**Adaptive measures based on workshop discussions**
	- The workshops were held when the Russian war of aggression against Ukraine had just started. No reactive adaptation measures were identified.
Proactive adaptation measures	- Ensuring that price of reindeer meat remains reasonable for example by enhancing the image of reindeer herding as a traditional nature-based livelihood (Economy and well-being of herders)<br>- Granting clear reindeer herding rights to secure the livelihood among competing land uses (Participation; Grazing peace, Available pastures).<br>- Emphasizing the contributions of reindeer herding to food security (Local knowledge).
**SSP4**	**Adaptive measures based on workshop discussions**
Reactive adaptation measures	- Increasing mutual understanding and collaboration between livelihoods, enabling coping with one another (Participation).)<br>- Dedicated person in RHCs who would manage the relationships between reindeer herding and other land uses within the RHC area (Participation).
Proactive adaptation measures	- Reindeer herders recognized as rights-holders; herders can genuinely influence land use in their home area (Participation).<br>- Legislative changes regarding number of reindeer, compensations and subsidies, land use, and rights to use the land (Participation; Economy and well-being of herders).<br>- Tools to ensure social equity in land use planning (e.g. strengthening the negotiation requirement with herders in Reindeer Herding Act); these would enhance also the predictability of land-use developments (Participation).

	- Reducing logging in state-owned lands in the reindeer herding area (Available pastures; Grazing peace; Economy and well-being of herders).<br>- Developing socio-cultural impact assessment of land-use developments in addition to Environmental Impact Assessments (Participation; Local knowledge).<br>- Developing land-use regulation towards better recognition of reindeer herding and customary rights of reindeer herders (Participation).<br>- Holistic land-use planning by state-based organizations, to understand the combined and cumulative impacts of various land uses on reindeer herding (Participation; Local knowledge).
**SSP5**	**Adaptive measures based on workshop discussions**
Reactive adaptation measures	- Catastrophe fund funded by the RHCs, municipalities and government enabling fast reaction to difficult weather and snow conditions (Economy and well-being of herders; Welfare of reindeer; Local knowledge)<br>- Emergency feeding with external help (e.g. defence forces, like in Norway). (Economy and well-being of herders; Welfare of reindeer; Communality).
Proactive adaptation measures	- Restoration of forest and wetland pastures to allow pasture rotation and flexibility in seasonal pasture us, important during difficult weather and snow conditions (Participation; Available pastures; Local knowledge)<br>- Enough resources for herders to plan the community-led adaptation (Economy and well-being of herders; Local knowledge; Communality).<br>- Monitoring system to anticipate difficult conditions (e.g. conditions on pastures, availability of forage and condition of reindeer in the autumn) (Participation; Local knowledge).

Table S3. General criteria for polysolutions, clusters, and related statements informing preferable adaptation measures.

General criteria	Sub-criteria	Participants' statements voiced in the workshops informing preferable adaptation measures
Depth: to secure herder access to land	Holistic values in reindeer herding	1. It is important to recognize also other than monetary values and meanings of reindeer herding, to value the "soul" of herding (Workshop 1, feedback session).<br>2. Open discussion on diverse values linked to land use is needed (Workshop 1, synthesis).<br>3. The valuation of reindeer herding among other land users, decision-makers and public needs to be increased (Workshop 1, synthesis; Workshop 2, group 1).<br>4. It would be important that values, identity, culture and tradition of herders would endure in the future despite the compromises with other land users (Workshop 2, group 1).
	Grazing peace and land-use planning	5. Land-use planning and governance are the keys to enhancing the continuity of reindeer herding (Workshop 1, feedback session)<br>6. It is important to increase herders' opportunities to influence land-use planning but reconciliation of different land uses is not always possible (Workshop 1, synthesis)<br>7. To safeguard the "grazing peace" of reindeer by means of land-use planning is important (Workshop 1, group 1).<br>8. To cherish the "holy trinity" (winter pastures, pasture rotation, and targeted feeding of reindeer) is essential (Workshop 2, group 1)<br>9. Stress is a common reason for reindeer deaths: grazing peace needs to be guaranteed (Workshop 2, group 2).
	Rights of reindeer herders	10. To recognize the right to herd reindeer is essential in land-use planning (Workshop 1, synthesis).<br>11. To better recognize Indigenous Sámi rights is important (Workshop 1, group 2; Workshop 2, group 2).<br>12. Indigenous Sámi rights need to be connected to concrete land-use rights (Workshop 2, group 1).<br>13. Free grazing right and "grazing peace" of reindeer form the basis of reindeer herding. It is the task of legislation to ensure these, to secure the customary intergenerational rights of herders (Workshop 1, group 4).<br>14. Land-use planning, legislation and Indigenous rights are affecting reindeer herding profitability, and "soul" of the livelihood through land use (Workshop 1, group 1).

		15. The requirement to negotiate with reindeer herders mentioned in the Reindeer Husbandry Act should be extended so that the herders would not have to listen to ready-made decisions (Workshop 2, group 1).<br>16. Traditional customary rights should be recognized to secure rights to herd reindeer (Workshop 2, group 2).
Width: managing cumulative impacts	Managing the cumulative impacts of other land uses on reindeer herding	17. A special certificate for reindeer products can enhance the positive image of reindeer herding among the general public and other land users (Workshop 1, Feedback session)<br>18. A certificate for other land users, gained by taking reindeer herding into account ("Reindeer friendly certificate") would also help (Workshop 1, feedback session).<br>19. To better recognize the socio-cultural impacts of land use on reindeer herding is essential (Workshop 1, synthesis)<br>20. Enhancing holistic land-use planning is needed (Workshop 1, synthesis).<br>21. Managing cumulative impacts should be a task of the state: one disturbance is ok, but when they multiply, it is a problem (Workshop 2, group 2).<br>22. Land-use planning is a key issue defining the future of reindeer herding (Workshop 2, group 1)<br>23. Setting clear "rules of the game" for other land users is important (Workshop 1, synthesis).<br>24. Cumulative impacts of other land uses change the possibilities for pasture rotation (Workshop 1, group 2).<br>25. Most of the challenges faced by reindeer herding connect in practice to land-use planning (Workshop 2, group 1).<br>26. Defending herding continuously against other land users is burdening and affects herders' well-being (Workshop 2, group 1).<br>27. The land-use negotiations should include all land-use projects; it is pointless to discuss them in isolation from each other (Workshop 2, synthesis).<br>28. Reindeer herding must be always secured when planning new land uses (Workshop 2, group 2).<br>29. The biggest issue: other land use is increasing and it is always having negative impacts on reindeer herding: this cannot continue forever (Workshop 2, group 2).<br>30. There is often pressure to reduce the number of reindeer, but all other land use is simultaneously increasing (Workshop 2, group 2).
	Connections to policy	31. It would be important that green transition projects (e.g. wind power, mining) be socially just for reindeer herders (Workshop 2, groups 1 & 2).

		32. To revitalize pastures by connecting to EU's Nature Restoration Law is a future opportunity (Workshop 2, synthesis).<br>33. There should be an environmental subsidy, which RHCs could apply to start restoration projects (Workshop 2, synthesis)<br>34. Development of subsidies and supporting policies to enhance profitability of reindeer herding also in the changing future are needed (Workshop 2, synthesis).
Pace: urgency of adaptation solutions	Connection between the welfare of reindeer and the well-being of herders	35. Well-being of reindeer and herds are the key to everything and connect to economy and well-being of herders (Workshop 2, group 1).<br>36. Continuity of the livelihood is connected to climate change, pastures, herders' rights and welfare of reindeer, also impacted by climate change (Workshop 2, group 1).<br>37. "If reindeer are not well, the reindeer herder is not feeling well either" (Workshop 1, group 1).
	Urgent need to improve profitability to secure the continuity of the livelihood	38. If there are no herders in the future, the intergenerational chain breaks (Workshop 2, groups 1 & 2).<br>39. No one from the younger generation will continue reindeer herding if they cannot get subsistence out of it (Workshop 2, group 1).<br>40. Difficult winters have increased in frequency during recent years, increasing the costs and affecting the profitability of reindeer herding (Workshop 1, group 2)<br>41. Reindeer feed is expensive especially in areas which lack fields for growing hay (Workshop 1, group 1).<br>42. If the reindeer herding livelihood is not profitable, it is difficult to get young herders to continue the profession (Workshop 2, main finding).<br>43. If profitability is weak, the next generation is not motivated to continue in the livelihood (Workshop 1, session 1, group 4; Workshop 2, group 2).<br>44. Many factors (e.g. other land uses, predators) affect the vitality of the reindeer herding livelihood, and through that, the intergenerational continuity of the livelihood (Workshop 2, group 1).

	Chronic crisis calling for solutions	45. Reindeer herding is currently in a situation of "chronic crisis" (Workshop 1, session 1, group 4).<br>46. Climate change increases the pressure to feed the reindeer, and when the cost of feed is simultaneously increasing, this forms a vicious cycle compromising the profitability of herding (Workshop 1, group 1).<br>47. With the support of positive measures (e.g. in land-use planning) reindeer herding could face the future without crises, or at least be viable despite the crises (Workshop 1, main finding).<br>48. It would be much easier for herders if the predator populations were better managed and controlled (Workshop 2, group 1).
Proactiveness: anticipating future crises	"Climate friendly certificate"	49. A "Climate friendly" brand for reindeer products could help to market the products (Workshop 1, feedback session).
	Proactive measures to prepare for difficult winters	50. Reindeer herding needs a dedicated climate change adaptation plan (Workshop 1, feedback session).<br>51. Climate change is the central cause of uncertainty among reindeer herders (Workshop 2, group 1)<br>52. Easy-to-use monitoring systems are needed for reindeer herding (Workshop 1, feedback session).<br>53. It would be important to be able to anticipate the difficult winters by proactive assessments (e.g. according to the mushroom yield in the autumn or whether the pastures are frozen) (Workshop 1, feedback session).<br>54. Climate change adaptation can be supported by "spare pastures" not used during easy winters (Workshop 2, synthesis)
	Court cases that work as precedents for future land-use struggles.	55. Enhancing other means than court cases to influence land-use planning is needed (Workhsop 1, synthesis).<br>56. Example court cases are important for the ability of reindeer herders to influence land-use planning (Workshop 1, feedback session).
Collaboration: adapting together	Importance of trust between land users	57. Lack of trust among land users is a challenge. There is a need for open discussions and interpretation of situations together (Workshop 1, feedback session)<br>58. Trust takes a long time to build but can be broken in an instant (Workshop 1, group 4).<br>59. Trust between stakeholders is a key for land-use planning (Workshop 2, groups 1 & 2)

	Need to collaborate with diverse land users	60. Reconciliation of different land uses is difficult due to context-specific situations (Workshop 1, feedback session).<br>61. More understanding and collaboration skills are needed between different land uses (Workshop 1, feedback session).<br>62. Motivation to find common understanding can unite different land users (Workshop 1, feedback session).<br>63. There is need for in-depth discussions and collaboration (Workshop 1, feedback session)<br>64. Further development of collaboration is needed between land users (Workshop 1, synthesis)<br>65. Timing of other land-use activities needs to be agreed with reindeer herders (e.g. not to disturb round-ups) (Workshop 2, group 1).<br>66. Natural resource planning must keep on moving towards more collaborative mechanisms (Workshop 2, synthesis).
	More resources for reindeer herders to enable collaboration	67. More resources for reindeer herders are needed to adapt and to influence land-use planning (Workhsop 1, synthesis)<br>68. More resources for reindeer herders are needed, to support collaboration (Workshop 1, group 3).<br>69. Participation fatigue caused by several simultaneous land-use planning processes is a problem (Workshop 2, synthesis).<br>70. Participation fatigue: every meeting repeats the same issues, and reindeer herders are always on the losing side (Workshop 2, group 2).
	Collaboration among reindeer herders	71. Communality among reindeer herders is important (Workshop 1, synthesis)<br>72. Land use contradictions with other land users may compromise the communality and even divide local communities (Workshop 1, group 3).<br>73. Without communality among herders within RHCs, the livelihood would not work (Workshop 2, group 1).<br>74. Communality provides resources for participating in land-use negotiations (Workshop 2, synthesis).
	Traditional knowledge	75. Traditional knowledge of Indigenous Sámi herders is important to recognize (Workshop 2, group 1)<br>76. Local traditional knowledge is a source of innovation (Workshop 1, group 3).<br>77. What happens to traditional herders' knowledge in the context of climate change (can it offer solutions in the changing environmental context)? (Workshop 2, group 1).<br>78. Traditional knowledge and values are the basis of the reindeer herding livelihood (Workshop 2, group 2).<br>79. New reindeer herders are raised to the livelihood from a young age onwards (Workshop 2, group 2).
